# CXCL14 and MCP1 are potent trophic factors associated with cell migration and angiogenesis leading to higher regenerative potential of dental pulp side population cells

**DOI:** 10.1186/s13287-015-0088-z

**Published:** 2015-05-29

**Authors:** Y Hayashi, M Murakami, R Kawamura, R Ishizaka, O Fukuta, M Nakashima

**Affiliations:** Department of Dental Regenerative Medicine, Center of Advanced Medicine for Dental and Oral Diseases, National Center for Geriatrics and Gerontology, Research Institute, Morioka 7-430, Obu, Aichi 474-8511 Japan; Department of Pediatric Dentistry, School of Dentistry, Aichi-Gakuin University suemoridouri 2-11, Nagoya, Aichi 464-8651 Japan; Department of Gerodontology, School of Dentistry, Aichi-Gakuin University suemoridouri 2-11, Nagoya, Aichi 464-8651 Japan

## Abstract

**Introduction:**

The release of trophic factors from mesenchymal stem cells (MSCs) is critical for tissue regeneration. A systematic investigation of the regenerative potential of trophic factors from different MSCs, however, has not been performed. Thus, in the present study, the regenerative potential of conditioned medium (CM) from dental pulp, bone marrow, and adipose tissue-derived CD31^−^ side population (SP) cells from an individual source was compared in an ectopic tooth transplantation model.

**Methods:**

The tooth root transplantation in an ectopic site model was used for investigation of the regenerative potential and trophic effects in vivo. Either pulp CD31^−^ SP cell populations (1×10^6^ cells) at the third to fourth passage or 5 μg/ml of CM from dental pulp, bone marrow, and adipose stem cells from four different individuals were injected into the root with collagen TE. Each root was transplanted subcutaneously in 5-week-old severe combined immunodeficiency mice. Each root with surrounding tissue was harvested for histology on days 7, 21, and 28 and for Western blot analysis and real-time reverse transcription-polymerase chain reaction (RT-PCR) analysis on day 28. Furthermore, the trophic factors responsible for the regenerative potential were identified as the upregulated genes present in pulp CD31^−^ SP cells when compared with the genes in both bone marrow and adipose CD31^−^ SP cells by using microarray analysis, real-time RT-PCR, and Western blot analysis.

**Results:**

Transplantation of pulp CM yielded increased volume of pulp regeneration, more bromodeoxyuridine (BrdU)-positive migrated cells, and fewer caspase 3-positive cells in the regenerated pulp compared with the others. Pulp CM also demonstrated significantly increased cell migration, anti-apoptosis, and angiogenesis in C2C12 cells. Higher expression of *CXCL14* and *MCP1* in pulp SP cells suggested candidate trophic factors. The stimulatory effects on both migration and angiogenesis of CXCL14 and MCP1 were demonstrated in vitro. In the regenerated tissue, BrdU-positive migrated cells expressed *CXCR4* and *CCR2*, receptors for CXCL14 and MCP1, respectively.

**Conclusions:**

The higher regenerative potential of pulp SP cells may be due to potent trophic factors, including CXCL14 and MCP1, which promote migration and angiogenesis.

## Introduction

Mesenchymal stem cell (MSC) therapies hold great potential to treat a wide range of diseases and tissue defects, and over 350 clinical trials have been started. The efficacy of MSC transplantation in humans, however, has not been well established and this due in part to an incomplete understanding of the fate of transplanted MSCs and the lack of regulation on cell fate, especially cell migration and therapeutic impact [1]. It is essential to understand and regulate the fate of transplanted MSCs by exploring cell functions and cell-cell communication in their in vivo microenvironment (niche) for efficient regenerative therapy. The therapeutic functions of transplanted MSCs are not simple innate actions but rather a coordinated process involving the host microenvironment [2]. MSCs are site-regulated and secrete bioactive factors at various concentrations in response to local surrounding microenvironmental cues [3]. For example, bone marrow-derived MSCs form islands of bone tissue inside the bone defect area, whereas the fat content becomes significantly higher when the surrounding host calvarium contains a high content of fatty tissue. These findings indicate that cell fate decisions are regulated by extrinsic control factors in the immediate microenvironment [4]. Furthermore, MSCs derived from different tissues have been compared for regenerative capability for some experimental models, including ectopic tooth transplantation, cardiac diseases, calvarial defects, ischemic diseases, and cutaneous wounds, resulting in different efficacies [5–8]. MSCs, CD31^−^ side population (SP) cells from the dental pulp, are enriched in stem/progenitor cells and significantly stimulate angiogenesis/vasculogenesis [9]. Similarly, CD31^−^ SP cells from bone marrow exhibit enhanced cardiomyogenic differentiation and cardiac regeneration [10]. Pulp, bone marrow, and adipose CD31^−^ SP cells are able to be isolated from the same individual porcine mandible. Thus, we have compared the regenerative potential of pulp CD31^−^ SP cells with that of bone marrow and adipose CD31^−^ SP cells from the same individual in ischemic hindlimb models, ischemic cerebrum models, and ectopic tooth transplantation models, resulting in higher angiogenic, neurogenic, and regenerative potential [11]. These regenerated tissues were similar in quality and independent of the origin of the MSCs. Robust matrix formation, however, was shown in the tissue regenerated with adipose MSC transplant in ectopic tooth transplantation model [11]. Our preliminary study showed that after ectopic transplantation of demineralized and trypsin-treated teeth with injection of any type of MSCs from pulp, bone marrow or adipose resulted in no regenerated tissue. These results suggest that both the endogenous cells and the biochemical components in the tooth have an inductive microenvironment to regulate the transplanted MSCs.

The regeneration-promoting properties of the transplanted MSCs are attributed largely to secretion and release of trophic factors to promote tissue regeneration rather than direct differentiation into functional mesenchymal cells [12]. MSCs serve as a source of various growth factors, cytokines, chemokines, and proteinases [13] essential to cell survival (reduced apoptosis), angiogenesis/vasculogenesis, neurogenesis, and matrix remodeling [14–18]. The paracrine factors secreted by cells can accumulate in the conditioned medium (CM). The therapeutic effects of MSC-CM have been reported in several experimental models of tissue destruction, such as injuries in liver [19–21], kidney [22], and lung [23]; ischemic heart [24]; ischemic stroke [25]; bone defects [26]; periodontal disease [27]; and dermal wounds [28]. The potential properties of MSC-CM in healing/regeneration promotion have been addressed in vitro, including enhanced proliferation, migration, anti-apoptosis, anti-inflammation, angiogenesis, neuritogenesis, and neuroprotection [12, 21, 29–31]. In vivo, mobilization of endogenous stem cells by MSC-CM enhances bone regeneration [26]. The mechanisms by which MSC-CM provide benefits for tissue regeneration, however, are not well understood. In addition, there have been no systematic studies on the regenerative potential of MSC-CM from different tissues from the same individual using the identical isolation and culture methods for each cell source. Transplantation of teeth in ectopic sites for pulp/dentin regeneration is an efficacious model to compare the MSC-CM isolated from different tissues.

Our previous studies have demonstrated that transplanted pulp MSCs and CD31^−^ SP cells do not differentiate directly into endothelial cells, neuronal cells, or host cells but have profound trophic effects on the nearby endogenous stem/progenitor cells in the ectopic tooth transplantation model [11]. The trophic effects of CM from pulp CD31^−^ SP cells are superior in vitro to CM from bone marrow and adipose CD31^−^ SP cells, leading to higher angiogenic and regenerative potential of transplanted pulp CD31^−^ SP cells in vivo compared with that of bone marrow and adipose CD31^−^ SP cells [11]. Thus, in the present study, to clarify the precise mechanisms of MSC-CM therapeutic effects, CM derived from porcine dental pulp, bone marrow, and adipose CD31^−^ SP cells from the same individual pig were evaluated in vivo for their pulp/dentin regeneration potential, promotion of vasculogenesis/angiogenesis, proliferation, and migratory and anti-apoptotic effects on surrounding endogenous cells. Furthermore, upregulated genes in pulp CD31^−^ SP cells compared with that of bone marrow and adipose CD31^−^ SP cells were identified as candidate trophic factors by microarray analysis and real-time reverse transcription-polymerase chain reaction (RT-PCR). The functional roles of the candidate factors and their cognate receptors in pulp regeneration were also investigated in vivo by immunohistochemistry and molecular biological approaches and in vitro.

## Methods

All animal experiments were conducted by using the strict guidelines of the Animal Protocol Committees (authorization number: AGUD156) and DNA Safety Programs at both National Center for Geriatrics and Gerontology, Research Institute and Aichi-Gakuin University.

### Cell isolation and preparation of conditioned media

CD31^−^ SP cells from pulp, bone marrow, and adipose tissue were isolated from porcine mandible of the same individual pig as described previously [11]. These isolated CD31^−^ SP cells were plated on 35-mm collagen type I-coated dishes (AGC TECHNO GLASS CO., Shizuoka, Japan) in Dulbecco’s modified Eagle’s medium (DMEM) (Sigma-Aldrich, St. Louis, MO, USA) supplemented with 10 % fetal bovine serum (Invitrogen Corporation, Carlsbad, CA, USA). Adherent cells prior to 60 % confluency were harvested by 0.02 % EDTA and re-plated at a 1:4 dilution. The culture medium was switched to DMEM without serum at 50 % confluency at the fourth to fifth passage of culture. The CM from pulp, bone marrow, and adipose CD31^−^ SP cells were collected 24 h later, concentrated approximately 25-fold by using ultrafiltration unit (Amicon Ultra-15 Centrifugal Filter Unit) with a 3-kDa molecular weight cutoff (Ultracel-3 membrane) (Millipore, Billerica, MA, USA), and stored with proteinase inhibitors (Halt™ proteinase inhibitor cocktail EDTA-free, Thermo Scientific, Rockford, IL, USA) at −80 °C until use. The protein concentration of the CM was determined by BradfordUltra™ (Expedeon, Cambridge, UK).

### Model of tooth root transplantation in an ectopic site

The tooth root transplantation in an ectopic site model was used for investigation of the regenerative potential and trophic effects in vivo. Porcine second incisors were extracted and their roots were cut out, 6 mm in length, followed by enlargement, 2 mm in width and sealing of one end with Zinc Phosphate cement (Elite Cement) (GC, Tokyo, Japan). Either pulp CD31^−^ SP cell populations (1×10^6^ cells) at the third to fourth passage or 5 μg/ml of CM from dental pulp, bone marrow, and adipose stem cells from four different individuals were injected into the root with collagen TE (Nitta Gelatin, Osaka, Japan). Each root was transplanted subcutaneously in 5-week-old severe combined immunodeficiency (SCID) mice (CB17, CLEA, Tokyo, Japan) (*n* = 26 mice). Each root with surrounding tissue was harvested for histology on days 7, 21, and 28 (*n* = 4 mice per time point) and for Western blot analysis and real-time RT-PCR analysis on day 28 (*n* = 4 mice, respectively). Tooth roots with a phosphate-buffered saline (PBS) injection with collagen TE were also transplanted as a control (*n* = 2 mice) and were harvested on days 21 and 28 (*n* = 1 mouse per time point). The tooth roots labelled with bromodeoxyuridine (BrdU) (11299964001, Roche, Basel, Switzerland) on day 3 were harvested on day 7 (*n* = 4 mice).

For histology, the tooth roots were fixed in 4 % paraformaldehyde (Nakarai Tesque, Kyoto, Japan) at 4 °C overnight and embedded in paraffin wax (Sigma-Aldrich) after demineralization with Kalkitox™ (Wako, Osaka, Japan). The paraffin sections (5 μm in thickness) were stained with hematoxylin and eosin. Four sections at 150-μm intervals for four roots, each transplanted with pulp CD31^−^ SP cells and three different CM, were examined for relative amounts of regenerative tissue by capturing video images of the histological preparations under binocular microscopy (M 205 FA, Leica, Wetzlar, Germany). On-screen image outlines of newly regenerated tissue and the root canal were traced by using Leica Application Suite software, and the ratio of the regenerated areas to the root canal areas was calculated (*n* = 4 teeth). Cell density was analyzed after counterstaining with Hoechst 33342 (1:1000) on a BZ-9000 Biorevo fluorescence microscope (Keyence, Osaka, Japan). The numbers of Hoechst-positive cells to the regenerated area on days 21 and 28 were calculated in three sections of each tooth root (*n* = 4 teeth).

Immunohistological analyses with mouse anti-rat RECA1 (rat endothelial cell antigen 1) (Sanbio BV, Uden, The Netherlands) (1:500) with biotinylated horse anti-mouse Texas Red secondary antibody (Vector Laboratories, Burlingame, CA, USA) (1:200) were performed to determine the level of neovascularization. The ratio of the area of RECA1-positive newly formed capillaries to the regenerated area on day 28 was calculated in three sections of each tooth root (*n* = 4 teeth).

In situ hybridization was performed in the regenerated tissues on day 28 by using a marker for pulp, thyrotropin-releasing hormone-degrading enzyme (*TRH-DE*), to identify pulp tissue regeneration as described previously [11]. The sections were examined by confocal laser microscopy (TCS SP5 conventional inverted microscope, Leica), and three-dimensional structures were reconstructed by Leica Application Suite Advanced Fluorescence (LAS AF) software (Leica). The expression of TRH-DE was also confirmed by Western blot analysis as described previously [32] in the regenerated tissue 28 days after transplantation of pulp CD31^−^ SP cells and three CM (*n* = 4 teeth). Normal pulp tissue from the incisors of the SCID mice was used as a positive control (*n* = 4 teeth). Real-time RT-PCR analyses were further performed by using markers for pulp tissue, *Syndecan 3* and *TRH-DE*, in the regenerated tissues from each of the four root conditions 28 days after transplantation of CD31^−^ SP cells and each CM. The normal pulp tissue from the same animals was used as a control (*n* = 4 teeth).

Odontoblastic differentiation was assessed by in situ hybridization by using a marker for odontoblasts, *enamelysin*. Digoxigenin (DIG) signals were detected by using a Tyramide Signal Amplification system. The numbers of *enamelysin-*positive cells along the dentinal wall in the regenerated tissue on day 28 were counted in three sections of each tooth (*n* = 4 teeth) by LAS AF software by using confocal laser microscopy.

To examine extracellular matrix formation, three paraffin sections of each root (*n* = 4 teeth) on day 28 were immunostained by using rabbit anti-aggrecan (ab9942, abcam, Cambridge, UK) (1:500) and goat anti-rabbit Alexa 488-conjugated secondary antibody (1:200) followed by counterstaining with Hoechst 33342. The positive area relative to the regenerated area was analyzed by using a BZ9000 Biorevo fluorescence microscope.

The promotion of proliferation by pulp CD31^−^ SP cells and by three CM was also examined by immunohistological analyses using proliferating cell nuclear antigen (PCNA) (1:200) antibodies and biotinylated anti-rabbit IgG secondary antibody (Vector Laboratories) (1:200). The sections were developed with the ABC reagent (Vector Laboratories) by using the DAB chromogen. After staining, captured video images of the histological preparations were analyzed under binocular microscopy (M 205 FA, Leica). The number of PCNA-positive cells was counted in the surrounding tissues of the transplanted teeth, including adipose, fibrous, and muscle tissues, and in the regenerated tissues on day 7 in three sections of each root by LAS AF software (*n* = 4 teeth).

Furthermore, the migratory effect of pulp CD31^−^ SP cells and three CM on endogenous cells was investigated. BrdU (150 mg/kg) was injected intraperitoneally into the surrounding tissues of each tooth 3 days after transplantation in SCID mice (*n* = 4 mice). These mice were transcardially perfused with physiological saline, followed by fixation with 4 % paraformaldehyde in 0.1 M phosphate buffer (pH 7.4) under deep anesthesia 7 days after transplantation. The paraffin sections of the transplanted teeth with the surrounding tissues were immunostained with mouse anti-human BrdU antibody (11299964001, Roche) (1:1000), followed by a goat anti-mouse Texas Red-conjugated secondary antibody (1:200) after counterstaining with Hoechst 33342. The number of BrdU-positive cells in the regenerated pulp areas was calculated in three sections of each tooth (*n* = 4 teeth) by LAS AF software by using confocal laser microscopy.

Immunohistological analysis was performed by using a rabbit anti-human caspase 3 antibody (Cell Signaling Technology Inc., Danvers, MA, USA) (1:200) with a goat anti-rabbit Alexa 488-conjugated secondary antibody (ab150077, abcam) (1:200) to examine the anti-apoptotic effects. The ratio of caspase 3-positive cells to Hoechst 33342-positive total cells in the regenerated tissue on day 7 was calculated in three sections of each root (*n* = 4 teeth).

### Trophic effects of the conditioned media from pulp, bone marrow, and adipose CD31^−^ side population cells

Mouse embryonic muscle myoblast cells (C2C12) (DS Pharma Biomedical, Osaka, Japan) were cultured in DMEM supplemented with 10 % fetal bovine serum (FBS) for 24 h to analyze the enhanced effect of CM of pulp, bone marrow, and adipose CD31^−^ SP cells on proliferation. Then, the medium was changed into DMEM without serum, and each CM was added to the medium at a final concentration of 5 μg/ml protein. Cell numbers were measured by Tetra-color one (Seikagaku Kogyo, Tokyo, Japan).

Horizontal chemotaxis assay was performed by using TAXIScan-FL (Effector Cell Institute, Tokyo, Japan) as previously described [33] to examine the effect of CM on C2C12 cell migration.

C2C12 cells were grown in DMEM for 3 days and then incubated with 500 nM staurosporine (Sigma-Aldrich) in DMEM supplemented with each CM at a concentration of 5 μg/ml protein to assess effects on apoptosis by the CM. As a control, vascular endothelial growth factor A (VEGF-A) (Reliatech GmbH, Wolfenbüttel, Germany) at a final concentration of 5 μg/ml was added. The cells were harvested 8 h later, treated with both Annexin V-FITC (Roche Diagnostics) and propidium iodide (Sigma-Aldrich) for 15 min, and analyzed by flow cytometry.

The endothelial differentiation potential of the CM was assessed in C2C12 cells cultured for 21 days in DMEM containing 2 % FBS, 5 μg/ml heparin (Lonza, Muenchensteinerstrasse, Switzerland), 5 μg/ml ascorbic acid (Lonza), and 5 μg/ml hydrocortisone (Lonza) supplemented with 5 μg/ml of each CM from pulp, bone marrow, and adipose CD31^−^ SP cells. Immunocytochemical analyses were performed for anti-vascular endothelial (VE)-cadherin/CD144 (primary antibody, 1:50) (Acris, Herford, Germany), and the positive number of cells was measured on a BZ-9000 Biorevo fluorescence microscope (Keyence) after counterstaining with Hoechst 33342. In addition, C2C12 cells were seeded on matrigel (BD Biosciences, San Jose, CA, USA) in DMEM containing 2 % FBS, 5 μg/ml heparin (Lonza), 5 μg/ml ascorbic acid (Lonza), and 5 μg/ml hydrocortisone (Lonza) supplemented with 5 μg/ml of each CM. Network formation was observed 4 h after cultivation. The mean lengths of the networks of cords and tube-like structures were measured under an inverted microscope (Leica, 6000B-4, Leica Microsystems GmbH, Wetzlar, Germany) by using Suite V3 (Leica).

### Analyses of upregulated genes in pulp CD31^−^ side population cells

The trophic factors responsible for the regenerative potential were identified as the upregulated genes present in pulp CD31^−^ SP cells when compared with the genes in both bone marrow and adipose CD31^−^ SP cells by using microarray analysis. Biotinylated cRNAs were prepared in accordance with the standard Affymetrix protocol from 250 ng of total RNA isolated from the three CD31^−^ SP cells that were obtained from the same individual (Affymetrix Japan K.K., Tokyo, Japan) as described previously [11]. The nine upregulated genes—chemokine (C-X-C motif) ligand 14 (*CXCL14*) (accession number: AY308800), granulocyte colony-stimulating factor (*G-CSF*) (accession number: NM_213842), brain-derived neurotrophic factor (*BDNF*) (accession number: NM_214259), neuropeptide Y (*NPY*) (accession number: NM_001256367), interleukin 1α (*IL-1α*) (accession number: NM_214029), interleukin 6 (*IL-6*) (accession number: NM_214399), interleukin 8 (*IL-8*) (accession number: NM_213867), interleukin 16 (*IL-16*) (accession number: NM_213751), and monocyte chemotactic protein 1 (*MCP1*) (accession number: NM_214214)—were further examined by real-time RT-PCR using primers (Table 1). The expression levels of these genes in pulp CD31^−^ SP cells were compared with those in both bone marrow and adipose CD31^−^ SP cell populations after normalization to β-actin.

**Table 1:** Porcine and mouse primers for real-time reverse transcription-polymerase chain reaction

Gene name	Species		5′ ← DNA sequence → 3′	Product size	Accession number
*CXCL14*	Porcine	Forward	ACGGGTCCAAGTGAAGTGC	206 bp	AY308800
		Reverse	GGCGTTGTACCACTTGATGA		
*G-CSF*	Porcine	Forward	CTGGACTGGGAGGTAGTTG	97 bp	NM_213842
		Reverse	CTTAGGGTAGGGGTTCACTC		
*BDNF*	Porcine	Forward	TTCAAGAGGCCTGACATCGT	180 bp	NM_214259
		Reverse	AGAAGAGGAGGCTCCAAAGG		
*NPY*	Porcine	Forward	CTCCGCCTCTGATAGTCTGC	200 bp	NM_001256367
		Reverse	CACTTCCCATCACCACACAG		
*IL-1-a*	Porcine	Forward	CAAGGACAGTGTGGTGATGG	215 bp	NM_214029
		Reverse	CATCATTCAGGATGCAGTGG		
*IL-6*	Porcine	Forward	AGCAAGGAGGTACTGGCAGA	187 bp	NM_214399
		Reverse	CAGCCTCGACATTTCCCTTA		
*IL-8*	Porcine	Forward	GCATTCCACACCTTTCCAC	145 bp	NM_213867
		Reverse	CTTCTGCACCCACTTTTCC		
*IL-16*	Porcine	Forward	TCAGCAGCCAAGTGTCATC	197 bp	NM_213751
		Reverse	TCTCAGCTCCGAAAGGTTG		
*MCP1*	Porcine	Forward	TCTCCAGTCACCTGCTGCTA	185 bp	NM_214214
		Reverse	TCCAGGTGGCTTATGGAGTC		
*β-actin*	Porcine	Forward	CTCTTCCAGCCCTCCTTCCT	80 bp	AJ312193
		Reverse	ACGTCGCACTTCATGATCGA		
*Syndecan 3*	Mouse	Forward	TGGCTACCTTGGACACAGAG	181 bp	NM_011520
		Reverse	GACTCTGGAGTTGGGGTCTG		
*TRH-DE*	Mouse	Forward	CCAGCAGGCATCAACACTTA	205 bp	NM_146241
		Reverse	CCTGTCATCACTGCAAGTTA		
*CXCR4*	Mouse	Forward	CCATGGCTGACTGGTACTTT	210 bp	NM_009911
		Reverse	TCAGGAGGAGGGCTGGGATC		
*CCR2*	Mouse	Forward	ATTCTGGGCTCACTATGCTG	188 bp	NM_009915
		Reverse	TCACCCCAAAGGTAACTGTC		
*β-actin*	Mouse	Forward	AAGTACCCCATTGAACACGG	257 bp	NM_007393
		Reverse	ATCACAATGCCAGTGGTACG		

BDNF, Brain-derived neurotrophic factor; bp, Base pairs; CCR2, Chemokine (C-C motif) receptor 2; CXCL14, Chemokine (C-X-C motif) ligand 14; CXCR4, Chemokine (C-X-C motif) receptor 4; G-CSF, Granulocyte colony-stimulating factor; IL, Interleukin; MCP1 (CCL2), Monocyte chemotactic protein 1 (chemokine (C-C motif) ligand 2); NPY, Neuropeptide Y; TRH-DE, Thyrotropin-releasing hormone-degrading enzyme

### Expression of trophic factors in CD31^−^ side population cells analysis by Western blot analysis

Expression of the upregulated genes, MCP1 and CXCL14 in pulp, bone marrow, and adipose CD31^−^ SP cells, was further analyzed by Western blot. Each cell population was lysed in 1 × SDS sample buffer (50 mM Tris-HCl, pH 6.8, 2 % SDS, 10 % glycerol, 6 % β-mercaptoethanol, and protease inhibitor) and boiled at 95 °C for 5 min. Protein (5 μg) from each cell lysate was electrophoresed and transferred onto Immobilon-P PVDF (polyvinylidene difluoride) membranes (Millipore). After blocking with 5 % skim milk (Wako, Osaka, Japan) in PBS-T at room temperature for 1 h, the membranes were first incubated with anti-CXCL14 (ab137541, abcam) antibody at 4 °C overnight and then with anti-rabbit IgG horseradish peroxidase (HRP)-linked secondary antibody (Cell Signaling, Beverly, MA, USA) at 4 °C overnight. The protein bands were detected by Luminate™ Forte Western HRP Substrate (Millipore). Images of the protein bands were captured by using the Light-Capture II cooled CCD (charge-coupled device) camera system (Atto Corp., Tokyo, Japan). After the CXCL14 protein band was captured, the membranes were incubated in Restore™ PLUS Western Blot Stripping Buffer (Thermo Scientific, Rockford, IL, USA) at room temperature for 15 minutes to remove antibody and were blocked by 5 % skim milk in PBS-T. Then the membranes were incubated with anti-MCP1 (ab7202, abcam) antibody at 4 °C overnight and then with anti-rabbit IgG HRP-linked secondary antibody at 4 °C overnight. The protein bands were detected and captured in the same way. As an internal control, the antibodies were removed from membrane again in the same way and incubated with anti-β-actin (RB-9421, NeoMarkers, Fremont, CA, USA) antibody and anti-rabbit IgG HRP-linked secondary antibody. The protein band detection and capture were performed in the same way. The intensity of each band was quantified by CS Analyzer version 3.0 (Atto Corp.). Relative protein expression level was evaluated on the basis of band intensity of CXCL14/β-actin and MCP1/β-actin. Each piece of data of Pulp SC was defined as 1.0.

### Expression of trophic factors and their receptors in regenerated tissues

Double-immunostaining of either CXCL14 or MCP1 with either glutamic-oxaloacetic transaminase (GOT) or BrdU in the regenerated tissue was performed to examine the localization of CXCL14 and MCP1 in relation to endogenous cells migrating from the surrounding tissues. The sections on day 7 were incubated with either rabbit anti-human CXCL14 (1:500) (abcam, ab137541) or rabbit anti-human MCP1 (1:500) (abcam, ab7202) at 4 °C overnight and then incubated with a donkey anti-rabbit Alexa 594-conjugated secondary antibody (1:200) (abcam, ab150076) at room temperature for 30 min. Then immunostaining for GOT (1:500) (LS Bio, LSB-24) with donkey anti-sheep Alexa 488-conjugated secondary antibody (abcam, ab150177) or BrdU antibody with a goat anti-mouse Alexa 488-conjugated secondary antibody (ab150117, abcam) (1:200) was carried out.

The localization of MCP1 with RECA1 in relation to newly formed blood vessels was also examined. The sections on day 7 were incubated with rabbit anti-human MCP1 at 4 °C overnight and then with a goat anti-rabbit Alexa 488-conjugated secondary antibody (1:200) at room temperature for 30 min, followed by immunostaining for RECA1 as described above. The diameter of 100 blood vessels selected at random was measured by LAS AF software, and the number of MCP1-positive cells in proximity to blood vessels was calculated in three sections of each tooth root (*n* = 4 teeth) by confocal laser microscopy. The mRNA expression of the receptors *CXCR4* and *CCR2* in the regenerated pulp tissues on day 28 was also examined by real-time RT-PCR (Table 1).

### Immunohistological and in situ hybridization analyses of the transplanted cells

Double staining of BrdU immunostaining and in situ hybridization of *CXCR4* and *CCR2* mRNA were performed to examine the expression of CXCR4 and CCR2 in cells migrating from the surrounding tissues. Mouse cDNAs for *CXCR4* (210 bp) and *CCR2* (188 bp) linearized with Spe I and Nco I, respectively, were used as anti-sense probes, and those linearized with Nco I and Spe I, respectively, were used as sense probes. The probes were constructed after subcloning of the PCR products by using the same primers as designed for real-time RT-PCR. Paraffin sections (5 μm each) of the regenerated tissues were immunostained with a mouse anti-human BrdU antibody (1:1000) at 4 °C overnight and then incubated with a goat anti-mouse Alexa 488-conjugated secondary antibody (1:100) at room temperature for 30 min to identify the migrated cells. Then the sections were treated with 20 μg/ml proteinase K for 3 min and hybridized with either *CXCR4* or *CCR2* probes in hybridization solution at 57 °C for 16 h. After treatment with RNase buffer at a concentration of 20 μg/ml, the sections were washed in high-stringency buffers, 2 × side scatter and 0.1 × side scatter, each for 20 min. The sections were then incubated with anti-DIG-HRP (Enzo Biochem, New York, NY, USA) after equilibration in Tris-NaCl-blocking buffer (pH 7.5) and developed in Rodamine Tyramide working solution (PerkinElmer, Boston, MA, USA) for 10 min. The sections were examined by confocal laser microscopy after counterstaining with Hoechst 33342 for 15 min. For the control sections, all procedures were processed in the same manner, but the primary antibodies were omitted and/or sense probes were used. The ratio of CXCR4-positive or CCR2-positive cells to BrdU-positive cells as determined by confocal laser microscopy in the regenerated pulp areas was calculated in three sections of each tooth (*n* = 4 teeth) by LAS AF software.

### Trophic effects of CXCL14 and MCP1 in vitro

Horizontal chemotaxis assay was performed by using TAXIScan-FL (Effector Cell Institute) with either mouse embryonic muscle myoblast cells (C2C12) (DS Pharma Biomedical) or human umbilical vein endothelial cells (HUVECs) to examine the effect of CXCL14 and MCP1 on cell migration as previously described [33]. Each cell fraction was placed into the single hole with which the device was held together with a stainless steel holder, and the following factors were placed into the contra-hole: either 1 μl of 1 μg/ml CXCL14 (866-CX-025, R&D Systems, Minneapolis, MN, USA), 3 ng/ml MCP1 (279-MC-010, R&D Systems), 5 μg/ml CM of pulp CD31^−^ SP cells, pulp CM with anti-CXCL14 (ab137541, abcam), pulp CM with 2.25 μg/ml anti-MCP1 (ab7202, abcam), 1 μg/ml CXCL14 with 60 μg/ml anti-CXCL14, or 3 ng/ml MCP1 with 2.25 μg/ml anti-MCP1 (ab7202, abcam).

Endothelial differentiation potential was assessed in HUVECs cultured for 14 days in DMEM containing 2 % FBS, 5 μg/ml heparin (Lonza), 5 μg/ml ascorbic acid (Lonza), and 5 μg/ml hydrocortisone (Lonza) supplemented with 5 μg/ml of CM of pulp CD31^−^ SP cells, 3 ng/ml of MCP1, 3 ng/ml MCP1 with 2.25 μg/ml anti-MCP1, or 1 μg/ml of CXCL14. Immunocytochemical analyses were performed for anti-VE-cadherin/CD144 (primary antibody, 1:50) (Acris, Herford, Germany), and the positive rate was measured on a BZ-9000 BIOREVO fluorescence microscope after counterstaining with Hoechst 33342.

### Statistical analyses

Data are reported as mean ± standard deviation. *P* values were calculated by using the Student’s *t* test and Tukey’s multiple comparison test in SPSS 21.0 (IBM Corporation, Armonk, NY, USA).

## Results

### Pulp regeneration after tooth root transplantation

**Fig. 1:**
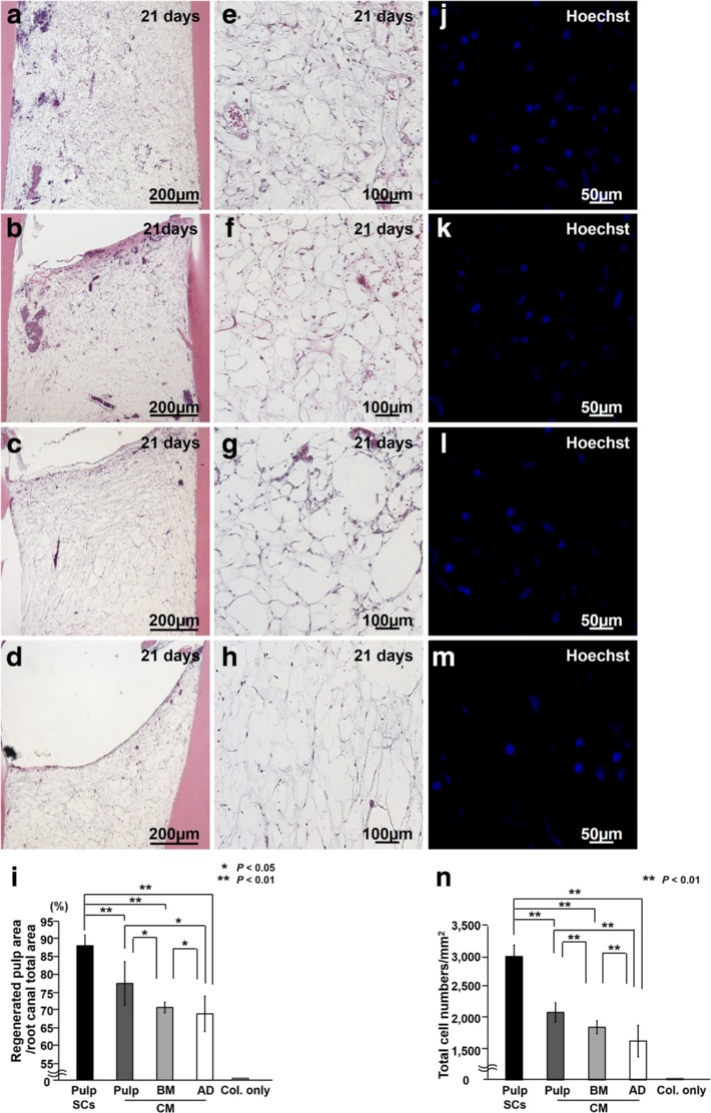
Pulp regeneration after ectopic tooth root transplantation in severe combined immunodeficiency mice. **a**, **e**, **j** Twenty-one days after transplantation of pulp CD31^−^ side population (SP) cells (Pulp SCs), (**b**, **f**, **k**) conditioned medium (CM) from pulp CD31^−^ SP cells (Pulp CM), (**c**, **g**, **l**) CM from bone marrow CD31^−^ SP cells (BM CM), and (**d**, **h**, **m**) CM from adipose CD31^−^ SP cells (AD CM). **a**-**h** Hematoxylin-and-eosin staining. **i** Ratio of regenerated area to root canal area on day 21. **j**-**m** Hoechst 33342 staining. **n** Cell density of the total regenerated area on day 21. Data are expressed as mean ± standard deviation of four determinations. *P < 0.05, **P < 0.01. Col. only, collagen only

The regenerative potential of the three CM of CD31^−^ SP cells from dental pulp, bone marrow, and adipose tissue was compared in a model involving tooth root transplantation to ectopic sites in SCID mice. Pulp-like tissue with well-organized vasculature was regenerated in the tooth root 21 days after pulp CD31^−^ SP cell transplantation (Fig. 1a, e). Similar pulp-like loose connective tissue was observed in transplants of the CM from pulp, bone marrow, and adipose CD31^−^ SP cells (Fig. 1b-d, f-h) as in pulp CD31^−^ SP cell transplants (Fig. 1a, e). The numbers of cells in the regenerated tissues stained with Hoechst 33342 was fewer in the three CM transplants (Fig. 1k-m) compared with the pulp CD31^−^ SP cell transplants (Fig. 1j). The histomorphometric analysis confirmed that the regenerated pulp area and cell density in the regenerated tissues by the three CM transplantation were significantly lower than those of the pulp CD31^−^ SP cell transplantation on day 21 (Fig. 1i, n) and day 28 (data not shown). Transplantation of the CM from pulp CD31^−^ SP cells yielded a larger regenerated area and higher cell density in the regenerated tissue compared with transplantation of the CM from bone marrow and adipose CD31^−^ SP cells on day 21 (Fig. 1i, n3). Bone marrow CM transplantation resulted in a significantly larger regenerated area and greater cell density compared with adipose CM transplantation on day 21 (Fig. 1i, n). There were no significant differences in the regenerated area and cell density between days 21 and 28 with pulp CD31^−^ SP cell transplants, although all three CM transplantations yielded a larger regenerated area and greater cell density on day 28 compared with that observed on day 21 (data not shown). These results suggest that pulp regeneration may not be completed 21 days after transplantation of CM, unlike pulp CD31^−^ SP cell transplantation, which is completed by day 21, and that pulp CM has higher trophic effects for pulp regeneration than other CM.

**Fig. 2:**
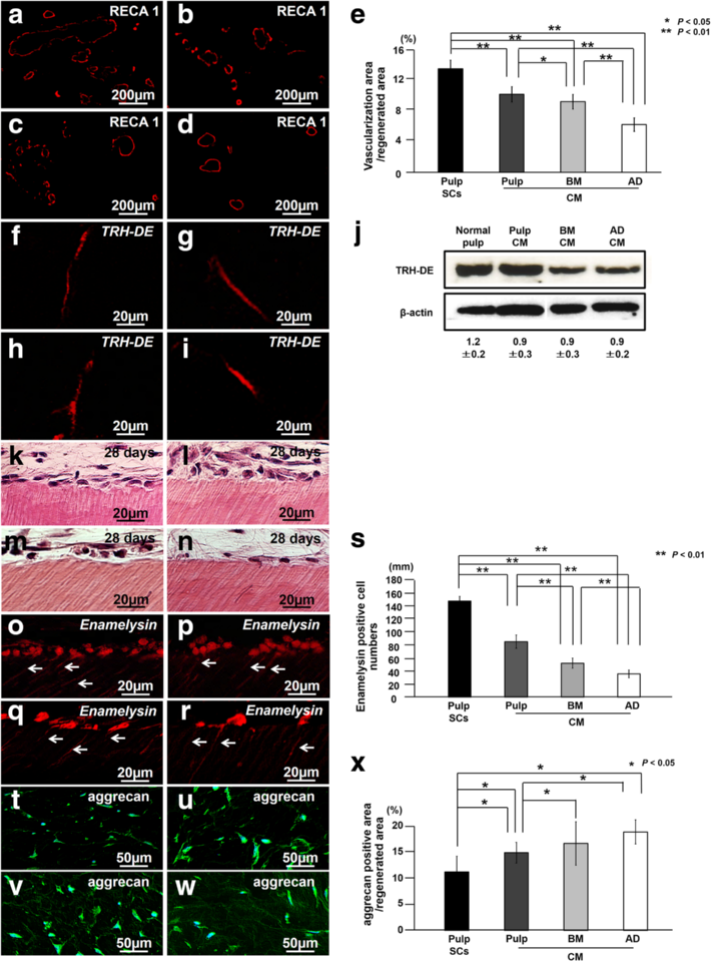
Characterization of regenerated tissue on day 28 in an ectopic tooth root transplantation model. **a**, **f**, **k**, **o**, **t** Transplant of pulp CD31^−^ side population (SP) cells (Pulp SCs). **b**, **g**, **l**, **p**, **u** Transplant of conditioned medium (CM) from pulp CD31^−^ SP cells (Pulp CM). **c**, **h**, **m**, **q**, **v** Transplant of CM from bone marrow CD31^−^ SP cells (BM CM). **d**, **i**, **n**, **r**, **w** Transplant of CM from adipose CD31^−^ SP cells (AD CM). **a**-**d** Immunostaining with rat endothelial cell antigen 1 (RECA1). **e** Ratio of vascularization area to the total regenerated area. (**f**-**i**) In situ hybridization analysis of expression of thyrotropin-releasing hormone-degrading enzyme (*TRH-DE*) as a pulp marker using an anti-sense probe reactive to both porcine and mouse genes. **j** Protein expression of TRH-DE in regenerated pulp after transplantation of CM from pulp, bone marrow (BM), and adipose (AD) CD31^−^ SP cells. **k**-**s** Odontoblastic differentiation potential in the regenerated pulp. **k**-**n** Odontoblastic cells along with the dentinal wall. **o**-**r** In situ hybridization analysis of *enamelysin*. Odontoblastic process extending into the tubular dentin (arrows). **s** Comparison of the numbers of *enamelysin*-positive cells along the dentinal wall. **t**-**w** Immunostaining with aggrecan (green) merged with Hoechst 33342 (Blue). **x** Ratio of aggrecan-positive area to the total regenerated area. Data are expressed as mean ± standard deviation of four determinations. *P < 0.05, **P < 0.01

Immunostaining with an RECA1 antibody revealed the neovascularization in the regenerated tissue by pulp CD31^−^ SP cell transplantation or three CM transplantations (Fig. 2a-d). Histomorphometric analysis demonstrated that neovascularization in the pulp CM transplants was much higher than that in both the bone marrow and adipose CM transplants on day 28 (Fig. 2e). *TRH-DE* mRNA and protein, a marker for pulp tissue, were similarly expressed in pulp CD31^−^ SP cell transplants and in all three CM transplants in the regenerated tissue as analyzed by both in situ hybridization and Western blotting (Fig. 2f-j). To further confirm that the regenerated tissues represent a pulp tissue phenotype, mRNA expression of both *Syndecan 3* and *TRH-DE*, specific markers for pulp, was examined by real-time RT-PCR analyses. Similar expression levels in the regenerated tissue to that in normal pulp tissue were observed (Table 2).

**Table 2:** Relative mRNA expression of pulp markers in regenerated tissue after transplantation of pulp CD31^−^ side population cells and pulp, bone marrow, and adipose conditioned medium

	Pulp	Pulp	BM	AD
	SC	CM	CM	CM
*Syndecan 3*	1.3±0.3	1.1±0.2	1.2±0.3	1.2±0.2
*TRH-DE*	1.2±0.2	1.1±0.1	0.9±0.4	1.0±0.5

AD, Adipose; BM, Bone marrow; CM, conditioned medium; SC, Side population cell; TRH-DE, Thyrotropin-releasing hormone-degrading enzyme

Odontoblast-like spindle-shaped cells attached to the dentinal wall, extending their processes for some distance into the dentin in the three CM transplants similar to CD31^−^ SP cell transplants (Fig. 2k-n). *Enamelysin-*positive odontoblast-like cells, however, were fewer in number in the bone marrow and in the adipose CM transplants when compared with that in the pulp CM transplants (Fig. 2o-r). Statistical analysis showed that the number of *enamelysin*-positive cells was significantly higher in the regenerated tissue in the pulp CM transplants compared with that in both the bone marrow and adipose CM transplants (Fig. 2s). Furthermore, enhanced matrix formation was observed in both the bone marrow and adipose CM transplants (Fig. 2t-w). Quantitative analysis indicated that the number of aggrecan-positive cells was significantly lower in the regenerated tissue in the pulp CM transplants compared with that in both the bone marrow and adipose CM transplants (Fig. 2x).

**Fig. 3:**
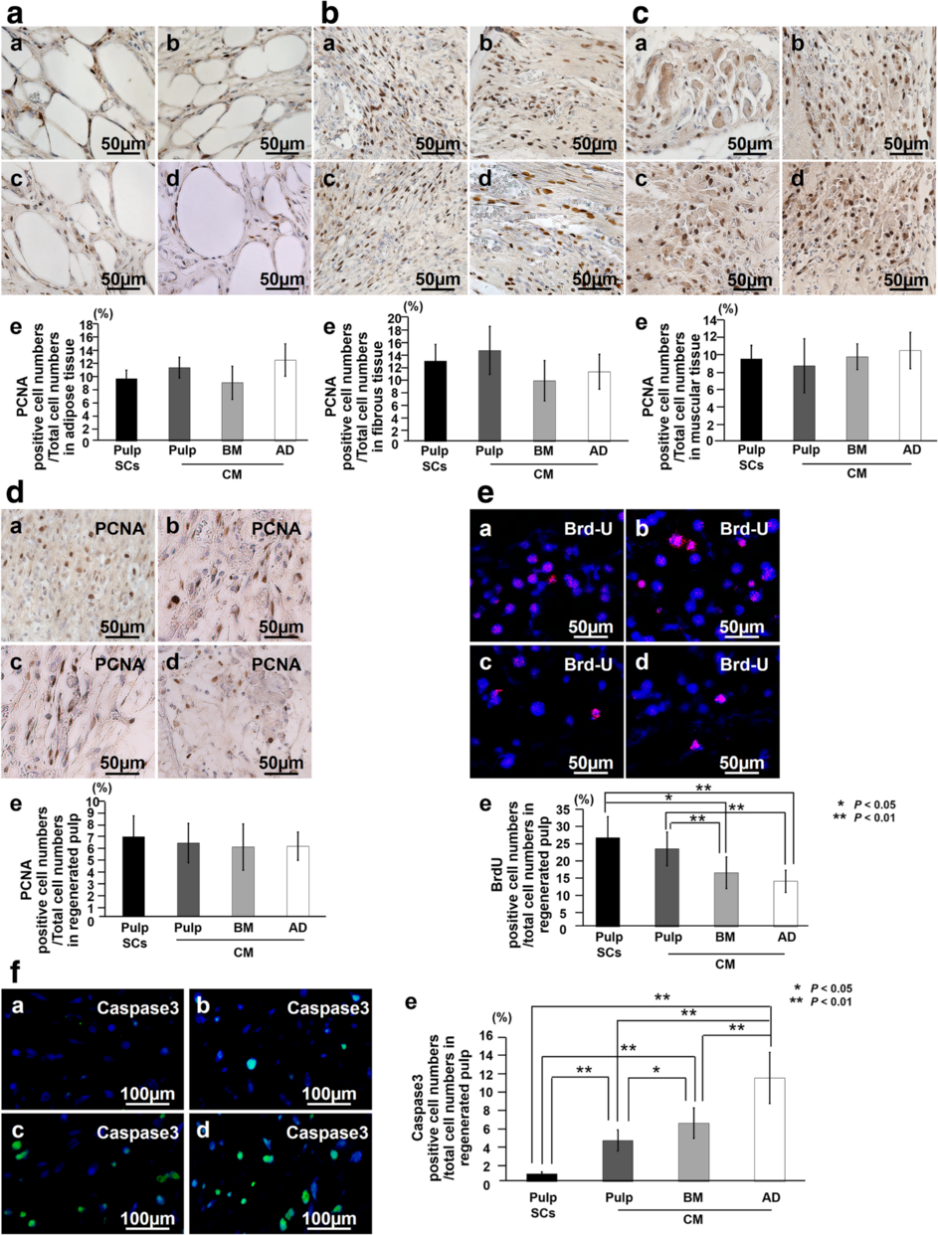
The effect of conditioned medium (CM) on proliferation, migration, and anti-apoptosis in the surrounding tissues of the transplanted teeth and the regenerated pulp. Immunostaining with proliferating cell nuclear antigen (PCNA) in adipose (AD) tissue (**a**), fibrous tissue (**b**), muscle tissue (**c**), and regenerated pulp tissue (**d**) 7 days after transplantation. (*a*) Pulp CD31^−^ side population (SP) cells, (*b*) CM from pulp CD31^−^ SP cells, (*c*) CM from bone marrow (BM) CD31^−^ SP cells, and (*d*) CM from AD of CD31^−^ SP cells. (*e*) Ratio of PCNA-positive cell numbers to the total cell numbers in the each tissue. **e** Bromodeoxyuridine (BrdU) (red) merged with Hoechst 33342 (Blue) in the regenerated pulp tissue on day 7. (*e*) Ratio of BrdU-positive cell numbers to the total cell numbers. **f** Immunostaining with caspase 3 (green) merged with Hoechst 33342 (Blue). (*e*) Ratio of caspase 3-positive cell numbers to the total cell numbers of regenerated pulp. Seven days after transplantation of (*a*) pulp CD31^−^ SP cells, (*b*) CM from pulp CD31^−^ SP cells, (*c*) CM from BM CD31^−^ SP cells, and (*d*) CM from AD of CD31^−^ SP cells. Data are expressed as mean ± standard deviation of four determinations. *P < 0.05, **P < 0.01

Proliferation in the regenerated tissue and in the surrounding tissues was examined on day 7 by immunostaining with PCNA. PCNA-positive cells were similarly detected in the surrounding tissues of the transplanted tooth, adipose, fibrous, and muscle tissues (Fig. 3a-c) and in the regenerated pulp (Fig. 3d). There was no significant difference in PCNA-positive cell numbers both in the surrounding tissues and in the regenerated tissue among the pulp cell transplants and the three CM transplants (Fig. 3a-d:e), suggesting no difference in the proliferative effect among the three CM.

The effect of CM on the migration of the endogenous cells from the surrounding tissues was also examined on day 7 after BrdU labeling for 4 days. Some of the cells stained with Hoechst 33342 were BrdU-labeled in the regenerated tissue (Fig. 3e). Pulp CM transplants yielded a greater number of BrdU-positive cells compared with that in both the bone marrow and adipose CM transplants, and the bone marrow CM transplants resulted in a significantly higher number of BrdU-positive cells compared with that in the adipose CM transplants (Fig. 3e:e), suggesting that pulp CM has the highest migratory effect among the three CM.

The anti-apoptotic activity of the CM was examined by immunostaining with caspase 3. Significantly fewer caspase 3-positive cells were observed in the tissue regenerated in the presence of pulp CD31^−^ SP cell transplanted cells compared with that in three CM transplants (Fig. 3f). Pulp CM transplants yielded fewer caspase 3-positive cells in the regenerated tissue compared with bone marrow and adipose CM transplants (Fig. 3f:e). These results demonstrated that transplantation of CM from pulp CD31^−^ SP cells yielded higher regenerated pulp tissue compared with CM from bone marrow and adipose CD31^−^ SP cells. Furthermore, this higher regenerative potential of CM from pulp CD31^−^ SP cells may be caused by its stimulatory effect on angiogenesis, migration, and anti-apoptosis.

**Fig. 4:**
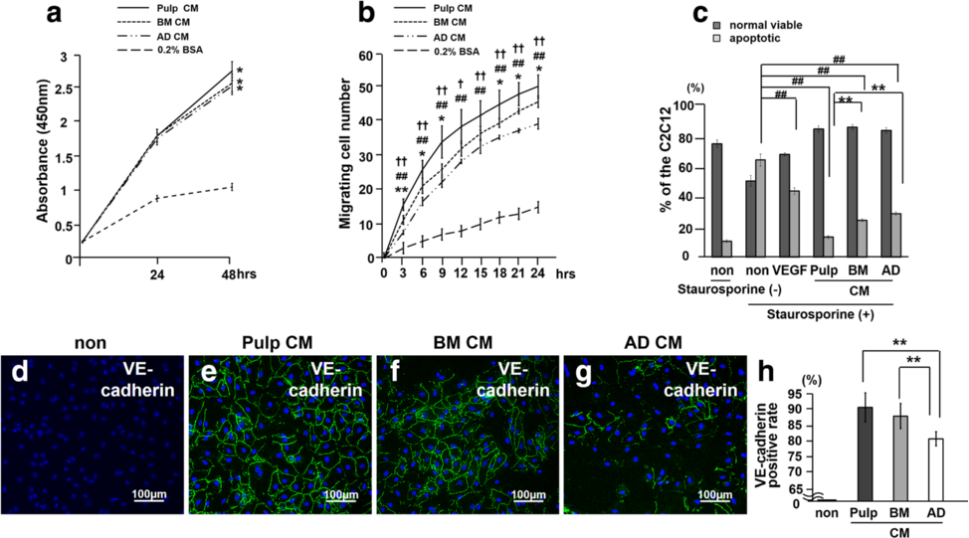
The trophic effects of conditioned medium (CM) of pulp, bone marrow, and adipose CD31^−^ side population (SP) cells in vitro. **a** The proliferative effect analyzed by using Tetra-color one® (*P < 0.01 versus 0.2 % bovine serum albumin (BSA)-treated). **b** The migratory effect analyzed by TAXIScan-FL® (*P < 0.05, **P < 0.01, versus adipose; ^†^ *P* < 0.05, ^††^ *P* < 0.01, versus bone marrow; ^##^ *P* < 0.01, versus 0.2 % BSA-treated). **c** The anti-apoptotic activities. The relative percentages of viable and apoptotic cells were analyzed by flow cytometry. Note the significantly higher anti-apoptotic effect of pulp CD31^−^ SP cells compared with CM from bone marrow and adipose CD31^−^ SP cells (^##^ *P* < 0.01, versus non-treated; **P < 0.01, versus pulp CM). **d**-**g** The angiogenic effect compared among the three CM analyzed by immunocytochemistry with vascular endothelial (VE)-cadherin. **h** Ratio of VE-cadherin-positive cell numbers to the total cell numbers. (**P < 0.01). Data are expressed as mean ± standard deviation of three determinations. The experiments were repeated three times. Only one representative experiment is presented. AD, Adipose; BM, Bone marrow; VEGF, Vascular endothelial growth factor

To confirm this hypothesis, the possible effects of CM from pulp, bone marrow, and adipose CD31^−^ SP cells on regenerative potential were further examined in vitro. The CM from the three CD31^−^ SP cells stimulated proliferation in C2C12 cells with similar efficacy (Fig. 4a). Pulp CM had increased migratory activity and higher anti-apoptotic activity compared with bone marrow and adipose CM (Fig. 4b, c). Furthermore, C2C12 differentiated into VE-cadherin-positive endothelial cells in the presence of each CM (Fig. 4e-g). Pulp CM had significantly greater stimulatory effects on the differentiation into endothelial cells than bone marrow and adipose CM (Fig. 4h), indicating that pulp CM had highest angiogenic potential. These in vitro findings demonstrate consistent results with all the in vivo effects of each CM in the ectopic tooth transplantation model.

### Expression of upregulated trophic factors and their receptors in the regenerated pulp tissue

**Fig. 5:**
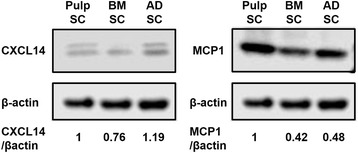
Western blot analyses of expression of trophic factors CXCL14 and MCP1 in pulp, bone marrow (BM), and adipose (AD) CD31^−^ side population (SP) cells (SCs). Relative protein expression level was evaluated on the basis of the band intensities of CXCL14/β-actin and MCP1/β-actin. Each piece of data of Pulp SCs was defined as 1.0. CXCL14, Chemokine (C-X-C motif) ligand 14; MCP1, Monocyte chemotactic protein 1

The transcriptomes of the three CD31^−^ SP cells were compared with each other by using a cDNA microarray analysis (Table 3). The highly upregulated genes in pulp CD31^−^ SP cells relative to bone marrow and adipose CD31^−^ SP cells included *CXCL14*, *G-CSF*, *BDNF*, *NPY*, *IL-1α*, *IL-6*, *IL-8*, *IL-16*, and *MCP1*. Real-time RT-PCR analysis for these genes further demonstrated that *CXCL14* and *MCP1* were expressed at higher levels in pulp CD31^−^ SP cells compared with the expression in both bone marrow and adipose CD31^−^ SP cells (Table 4). Higher expression of MCP1 protein was also demonstrated in pulp CD31^−^ SP cells compared with that in bone marrow and adipose CD31^−^ SP cells. Adipose CD31^−^ SP cells yielded a higher expression of CXCL14 protein compared with that in both the pulp and bone marrow CD31^−^ SP cells, and the pulp CD31^−^ SP cells resulted in a higher expression of CXCL14 protein compared with that in the bone marrow CD31^−^ SP cells. There was, however, no significant difference in expression level (Fig. 5). These results suggest that CXCL14 and MCP1 may be potential candidates for the prominent trophic factors of pulp CD31^−^ SP cells responsible for the increased regenerated tissue.

**Table 3:** Upregulated genes expressed in pulp CD31^−^ side population cells compared with bone marrow and adipose side population cells

	Gene name	Accession number	Pulp SCs/BM SC	Pulp SCs/AD SC
1 *CXCL14*	Chemokine -X-C motif ligand 14	AY308800	20	14
2 *G-CSF*	Granulocyte colony-stimulating factor	NM_213842	20	16
3 *BDNF*	Brain-derived neurotrophic factor	NM_214259	19	3.4
4 *NPY*	Neuropeptide Y	NM_001256367	6.6	8.6
5 *IL-1-a*	Interleukin 1-a	NM_214029	35	12
6 *IL-6*	Interleukin 6	NM_214399	2.6	3.5
7 *IL-8*	Interleukin 8	NM_21 3867	220	100
8 *IL-16*	Interleukin 16	NM_213751	12	3.5
9 *MCP1*	Monocyte chemotactic protein 1	NM_214214	2.3	1.8

AD, Adipose; BM, Bone marrow; SC, Side population cell

**Table 4:** Relative mRNA expression of the upregulated genes in pulp CD31^−^ side population cells compared with bone marrow and adipose side population cells

Gene name	Pulp SCs/BM SCs	Pulp SCs/AD SCs
1 *CXCL14*	93±02**	43±02^##^
2 *G-CSF*	1.9±0.1*	1.6±0.2
3 *BDNF*	1.6±0.1	1.2±0.1
4 *NPY*	1.9±0.2*	35±0.4^##^
5 *IL-1-a*	1.3±0.1	6.7±0.2^##^
6 *IL-6*	2.1±0.2*	46±02^##^
7 *IL-8*	53±03**	1.5±0.1
8 *IL-16*	53±02**	13±0.3^##^
9 *MCP1*	2.1±0.1*	4.4±0.1^##^

*P < 0.05, **P < 0.01: pulp side population cells (SCs) versus bone marrow (BM) SCs, ^##^ *P* <0.01: pulp SCs versus adipose (AD) SCs

**Fig. 6:**
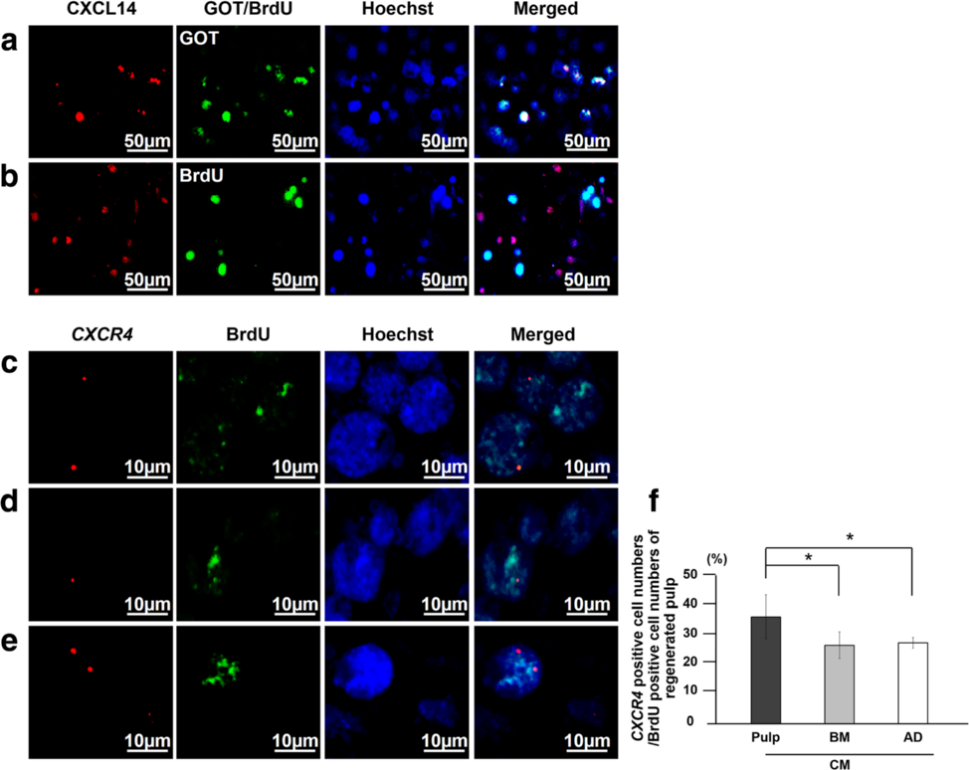
Localization of chemokine (C-X-C motif) ligand 14 (CXCL14) and its receptor, CXCR4, related to the transplanted cells and the endogenous migrated cells in the regenerated pulp on day 7. **a**, **b** Immunostaining of CXCL14 (red) merged with glutamic-oxaloacetic transaminase 2 (GOT2) or bromodeoxyuridine (BrdU) (green) and Hoechst 33342 (blue) after transplantation of pulp CD31^−^ side population (SP) cells. **c**-**e** In situ hybridization and immunohistochemical analysis of expression of C-X-C chemokine receptor type 4 (CXCR4) (red) merged with BrdU (green) after transplantation of conditioned media (CM) from pulp (**c**), bone marrow (**d**), and adipose CD31^−^ SP cells (**e**). **f** Ratio of CXCR4-positive cell numbers to BrdU-positive cell numbers in regenerated pulp tissues. Data are expressed as mean ± standard deviation of four determinations. *P < 0.05

**Fig. 7:**
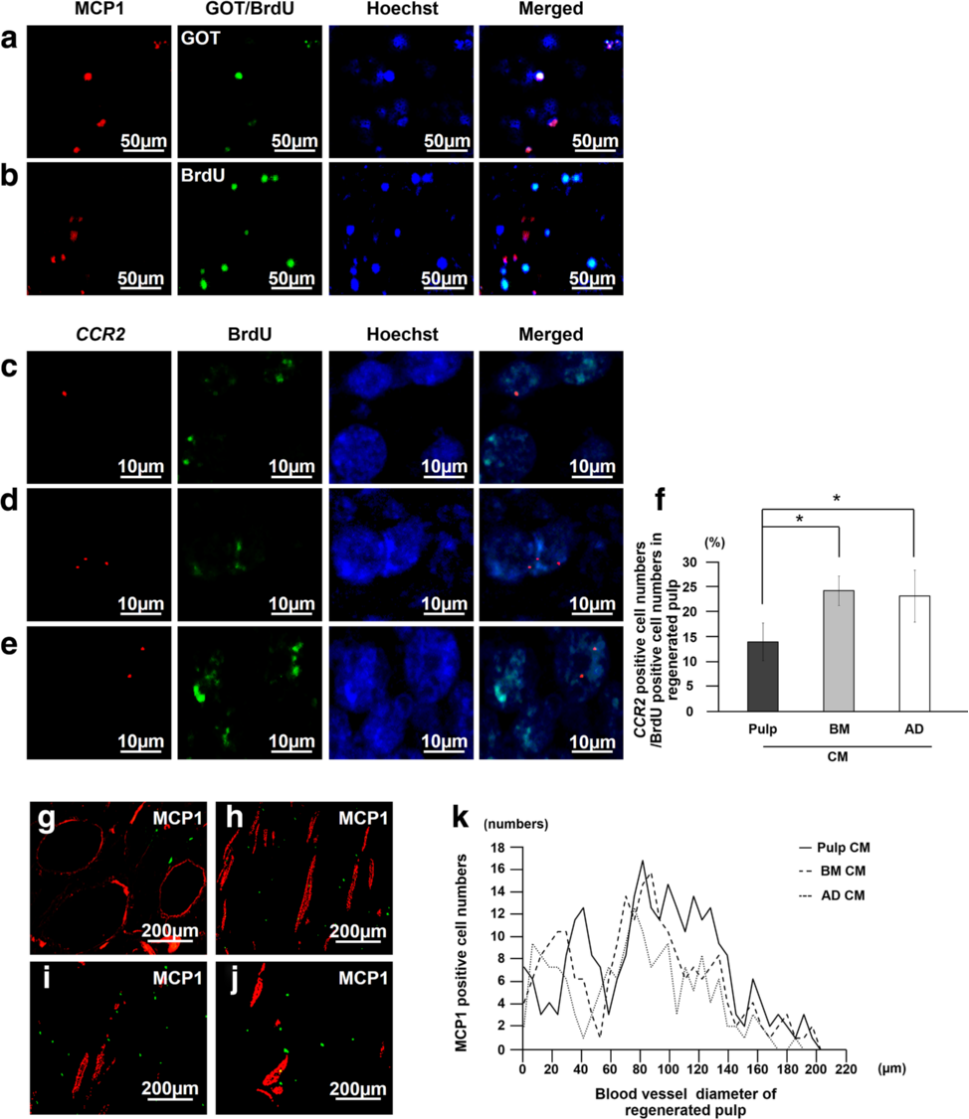
Localization of monocyte chemotactic protein 1 (MCP1) and its receptor, CCR2, related to the transplanted cells and the endogenous migrated cells in the regenerated pulp. **a**, **b** Immunostaining of MCP1 (red) merged with glutamic-oxaloacetic transaminase 2 (GOT2) or bromodeoxyuridine (BrdU) (green) and Hoechst 33342 (blue) 7 days after transplantation of pulp CD31^−^ side population (SP) cells. **c**-**e** In situ hybridization and immunohistochemical analysis of expression of CCR2 (red) merged with BrdU (green) 7 days after transplantation of conditioned media (CM) from pulp (**c**), bone marrow (**d**), and adipose CD31^−^ SP cells (**e**). **f** Ratio of CCR2-positive cell numbers to BrdU-positive cell numbers in the regenerated pulp. Data are expressed as mean ± standard deviation of four determinations. *P < 0.05. **g**-**j** MCP1-positive cells (green) in proximity to the newly formed vessels immunostained with rat endothelial cell antigen 1 (RECA1) (red) 28 days after transplantation of pulp CD31^−^ SP cells (**g**), CM of pulp (**h**), CM of bone marrow (**i**), and CM of adipose CD31^−^ SP cells (**j**). Note no co-localization. **k** The number of MCP1-positive cells related to the diameter of blood vessels

Recent studies have demonstrated that CXCL14 and MCP1 promoted cell migration and angiogenesis/vasculogenesis, respectively [34, 35]. Thus, localization of CXCL14 and MCP1 related to the migrated cells and to the newly formed vessels in the regenerated pulp tissue 7 days after transplantation of pulp CD31^−^ SP cells was examined. CXCL14- or MCP1-positive reactivity colocalized with GOT-positive transplanted cells (Figs. 6a and 7a) but did not colocalize with BrdU-positive migrated cells in the regenerated tissue after transplantation of pulp CD31^−^ SP cells (Figs. 6b and 7b), indicating that CXCL14 and MCP1 are secreted by the transplanted cells and not by the endogenous migrated cells. Confocal laser microscopic analysis demonstrated that MCP1-positive cells were not co-localized to vessels but were in proximity to two different sized vessels of approximately 40 μm and 70-130 μm in diameter (Fig. 7g-k), suggesting the trophic effect of MCP1 on angiogenesis, especially for arterioles and venules (small veins).

Next, the expression of each receptor for CXCL14 and MCP1, *CXCR4* and *CCR2*, respectively, was examined. The double-staining analysis by immunohistology and in situ hybridization in the regenerated tissue on day 7 demonstrated that migrated BrdU-positive endogenous cells expressed *CXCR4* (Fig. 6c-e) at significantly higher levels in pulp CM transplants when compared with the lower level in both bone marrow and adipose CM transplants (Fig. 6f). On the other hand, migrated cells expressed relatively reduced *CCR2* levels in the pulp CM transplants compared with the higher levels in both bone marrow and adipose CM transplants (Fig. 7f). The endogenous cells may migrate from the surrounding tissues by either the CXCL14-CXCR4 axis or MCP1-CCR2 axis with different rates among the three CM transplants. Expression of *CXCR4* and *CCR2* in the regenerated pulp tissues was also examined by real-time RT-PCR analysis 28 days after transplantation of the three CM, demonstrating significantly higher expression compared with normal pulp tissue (Table 5). Expression of *CXCR4* was significantly higher and expression of *CCR2* was significantly lower in the pulp CM transplants compared with bone marrow and adipose CM transplants, consistent with the findings of higher *CXCR4-*positive rate (Fig. 6f) and lower *CCR2-*positive cell rate in BrdU-positive cells (Fig. 7F). Thus, these results suggest the trophic effect of CXCL14 and MCP1 on the migrated endogenous cells through their receptors for migration and pulp regeneration.

**Table 5:** Relative mRNA expression of chemokine receptors in regenerated pulp after transplantation of pulp CD31^−^ side population cells and pulp, bone marrow, and adipose conditioned medium compared with normal pulp

Gene name	Pulp SC	Pulp CM	BM CM	AD CM
*CXCR4*	64±03**	6.7±0.4**^,††,§^	2.8±0.7*	3.8±1.2*
*CCR2*	1.7±0.3	53±0.6**	112±06**^,#^	8.2±0.4**

*P < 0.05, **P < 0.01: versus normal pulp, ^††^ *P* < 0.01: versus bone marrow (BM) conditioned medium (CM), ^§^ *P* < 0.05: versus adipose (AD) CM, ^#^ *P* <0.05: versus pulp CM. SC, Side population cell

#### Trophic effects of CXCL14 *and* MCP1 in vitro

**Fig. 8:**
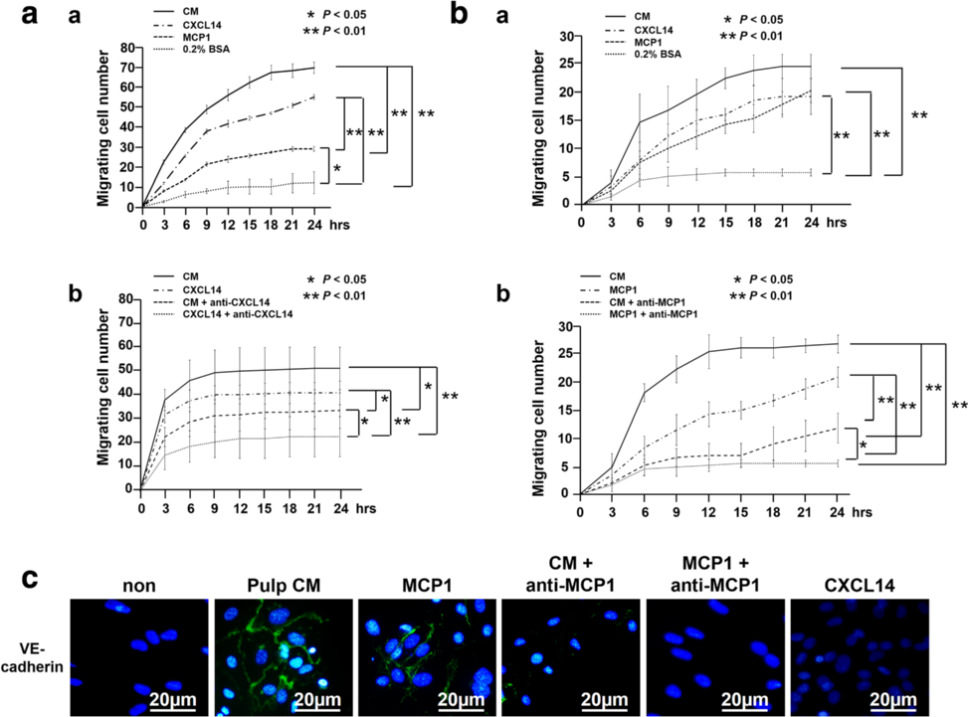
Trophic effects of CXCL14 and MCP1 compared with conditioned medium (CM) of pulp CD31^−^ side population (SP) cells in vitro. **a**, **b** Effect of CXCL14 and MCP1 on migration (*a*) and its inhibition of anti-CXCL14 (**a**) or anti-MCP1 (**b**) (*b*) analyzed by TAXIScan-FL in (**a**) C2C12 and (**b**) human umbilical vein endothelial cells (HUVECs). **c** Angiogenic effect of MCP1 analyzed by immunocytochemistry with vascular endothelial (VE)-cadherin. Relative percentages of viable and apoptotic cells in the presence of 500 nM staurosporine analyzed by flow cytometry. Data are expressed as mean ± standard deviation of four determinations. *P < 0.05, **P < 0.01. CXCL14, Chemokine (C-X-C motif) ligand 14; MCP1, Monocyte chemotactic protein 1

Next, we examined the migratory activity of CXCL14 and MCP1 and the angiogenic potential of MCP1 with the CM of pulp CD31^−^ SP cells in vitro. The migratory activity of CM, CXCL14, and MCP1 was significantly higher than that of the control in C2C12 cells. There was a significant difference in the migratory activity between CXCL14 and MCP1 (Fig. 8a:a), although there was no significant difference between the CM and CXCL14. The activity of either the CM or CXCL14 was significantly reduced when incubated together with anti-CXCL14 (Fig. 8a:b). HUVEC migration to the CM, CXCL14, and MCP1 was also significantly higher than that of the control, and there was no significant difference among the CM, CXCL14, and MCP1 (Fig. 8b:a). The migratory activity of either the CM or MCP1 was significantly reduced when incubated together with anti-MCP1 (Fig. 8b:b). These results demonstrate the stimulatory effect of CXCL14 and MCP1 on cell migration, although pulp CM may contain other trophic factors which promote cell migration. The angiogenic potential was further demonstrated by immunostaining with VE-cadherin on day 14 (Fig. 8c). Pulp CM and MCP1 similarly induced differentiation of HUVECs into an endothelial cell lineage positive for VE-cadherin. HUVECs were, however, negatively stained with VE-cadherin in the presence of anti-MCP1 either to pulp CM or to MCP1. These results suggest that the angiogenic potential of pulp CM may be partly attributed to MCP1.

## Discussion

MSCs from various tissues have attracted much attention for the treatment of many diseases and injuries, including bone and cartilage disorders, cardiovascular diseases, neuronal injury, and neurodegeneration via a variety of secreted trophic signals [36]. The trophic factors support regenerative processes in the damaged tissue, induce angiogenesis/neurogenesis, protect cells from apoptosis, and modulate the immune system [37]. Thus, the identification of the trophic factors applicable in regenerative medicine has recently become a subject of intense investigation. Proteomic techniques are currently used for the identification of trophic factors [38]. The transplanted MSCs and their trophic factors, however, are regulated by a complicated microenvironment consisting of stem cell niches, the endogenous stem/progenitor cells, and the biochemical and biophysical contributions of the extracellular matrix in vivo. Thus, it is essential to use an in vivo model for identification of candidate trophic factors for potential therapeutic applications. The tooth transplantation model in an ectopic site provides a stable microenvironment [39–41] and has been used to compare trophic factors, such as basic fibroblast growth factor (bFGF) and VEGF, for their regenerative potential with dental pulp MSCs [42, 43]. Our recent morphometric comparative study using the ectopic tooth transplantation model has demonstrated that MSCs from dental pulp, bone marrow, and adipose tissue qualitatively share properties in pulp regeneration but not quantitatively. The transplanted MSCs do not directly differentiate into endothelial cells, neural cells, or host pulp cells in the regenerated tissues but release a variety of trophic factors [11], suggesting that the amount of regenerated pulp tissues may depend on the concentration of trophic factors released from the different MSCs. Thus, in this investigation, to further clarify the trophic effects which highly influence the regenerative potential of MSCs, CM derived from porcine pulp, bone marrow, and adipose CD31^−^ SP cells from the same individual pig were compared in vitro and in vivo in the ectopic tooth transplantation model. We found that the CM of pulp CD31^−^ SP cells exhibited a significantly greater stimulatory effect on migration, anti-apoptosis, and angiogenesis and had little effect on cell proliferation compared with the non-pulp-derived CM in vitro. On the other hand, transplantation of the three CM demonstrated similar morphological features of the regenerated pulp tissue and similar expression of a pulp marker, TRH-DE, to transplants of pulp CD31^−^ SP cells, indicating pulp tissue regeneration. The BrdU-labeled cells derived from the surrounding tissues were detected in the regenerated tissues of all transplants, indicating migration of the endogenous cells in the tooth. These results suggested that pulp tissue may be regenerated by endogenous host cells that migrate in response to the microenvironment of the tooth in all CM transplants. Transplantation of pulp CM, however, produced a greater amount of regenerated pulp tissue and increased cell and capillary density compared with transplantation of non-pulp-derived CM. Thus, these results demonstrate that the distinct regenerative potential among the three CM may be due not to a proliferative effect but rather to migratory, anti-apoptotic, and angiogenic effects induced by trophic factors.

To identify candidate trophic factors responsible for the regenerative potential of MSCs, nine upregulated genes highly expressed in pulp CD31^−^ SP cells identified by microarray analysis were further analyzed by real-time RT-PCR, resulting in identification of *CXCL14*, *IL-6*, *IL-16*, *MCP1*, and *NPY* as potential mediators of the regenerative effect of pulp-derived CM*.* These three genes were expressed at significantly higher levels in pulp CD31^−^ SP cells compared with the levels observed in both bone marrow and adipose CD31^−^ SP cells. Higher expression of CXCL14 and MCP1 in pulp CD31^−^ SP cells was also confirmed by Western blot analysis. MCP1 have been previously identified in the MSC secretome [44], but CXCL14 was not previously identified.

CXCL14 is a member of the CXC chemokine family. The amino acid composition of CXCL14 is well conserved among various species. CXCL14 and CXCL12 (also known as SDF-1) are considered to be primordial or ancient chemokines on the basis of their sequence conservation among all classes of vertebrates and their homeostatic roles [45]. CXCL14 specifically binds to CXCR4 with high affinity and is representative of a molecule coevolved with the CXCL12/CXCR4 axis to modulate physiological processes [46]. CXCL12 and its receptor CXCR4 have critical functions in the migration of various types of MSCs [47, 48]. CXCL12, however, is secreted minimally by CD31^−^ SP cells [49]. Our study demonstrated that the CXCL14 gene and protein were expressed in CD31^−^ SP cells. Mouse embryonic muscle myoblast cell (C2C12) migration was stimulated by both pulp CM and CXCL14, and the enhanced migration was significantly reduced by CXCL14 antibody, indicating that pulp CM contains CXCL14 as a migration factor. In vivo CXCL14 was expressed in the regenerated tissue in the transplanted pulp CD31^−^ SP cells but not in the BrdU-positive endogenous cells that had migrated, clearly suggesting its trophic effect. The ratio of CXCR4-positive cells to BrdU-positive migrated cells in the regenerated tissue was significantly higher in pulp CM transplants when compared with that in bone marrow and in adipose tissue CM-treated transplants, suggesting prominent CXCL14-CXCR4 axis activity for endogenous cell migration in pulp CM transplants. Differentiation of HUVECs into endothelial cell lineage was not induced by CXCL14 in vitro. Thus, the present results suggest that CXCL14 may be an effective trophic factor which influences endogenous cell migration, resulting in increased cell density and regeneration after transplantation of MSCs or the CM of MSCs.

MCP1/CCL2 is a member of the C-C chemokine family. MCP1 is implicated in angiogenesis and promotes HUVEC capillary-like structure formation in vitro [50–53]. MCP1 affects the angiogenic process through increased expression of both VEGF and HIF-α (hypoxia-inducible factor 1-alpha) and through activation of the Ets-1 transcription factor, suggesting that its angiogenic properties might be mediated by secondary angiogenic factors [51, 52]. bFGF strongly stimulates the expression of MCP1 in mesenchymal cells but not in endothelial cells. In addition, the MCP1-CCR2 axis plays a critical biological role in recovery of blood flow in a murine hindlimb ischemic model, implicating the stimulatory function of MCP1 in adaptive and fibroblast growth factor-2-mediated therapeutic neovascularization [53, 54]. Secreted MCP1 mediates the angiogenic effect of tissue factor by recruiting smooth muscle cells toward endothelial cells and facilitates the maturation of newly formed microvessels in matrigel plugs in vivo [55]. In this investigation, higher expression of MCP1 was demonstrated in pulp CD31^−^ SP cells compared with that in bone marrow and adipose CD31^−^ SP cells in vitro. MCP1 stimulated HUVEC migration and angiogenesis, which were inhibited by anti-MCP1. On the other hand, MCP1 had a small stimulatory effect on C2C12 myoblast migration. In vivo MCP1 was expressed in the transplanted pulp CD31^−^ SP cells but not in the BrdU-positive endogenous cells that had migrated into the regenerated tissue. MCP1-positive cells were localized in proximity to both arterioles and venules (small veins). On day 7, the expression of CCR2 in the BrdU-labeled endogenous cells that had migrated was lower in the pulp CM transplants compared with that in the bone marrow and adipose CM transplants. These results suggest that the MCP1/CCR2 axis might have a regulatory role in both the migration of endothelial cells and maturation of neovascularization.

## Conclusions

Transplantation of the CM of pulp CD31^−^ SP cells yielded a larger volume of pulp regeneration, demonstrating increased migration of endogenous cells from the surrounding tissue, elevated angiogenesis, and decreased apoptosis in the regenerated pulp compared with the transplants of the CM from bone marrow and adipose CD31^−^ SP cells. Upregulated genes, including *CXCL14* and *MCP1*, in pulp CD31^−^ SP cells were compared with those in bone marrow and adipose CD31^−^ SP cells and were identified as candidate trophic factors. The stimulatory effects on migration and angiogenesis of CXCL14 and MCP1 were demonstrated in vitro. In the regenerated tissue, the migrated cells from the surrounding tissue expressed the receptors, *CXCR4* and *CCR2*. MCP1 was expressed in proximity to both arterioles and venules. These results demonstrate that potent factors, including CXCL14 and MCP1, may be associated with the higher functional regenerative potential of pulp CD31^−^ SP cells with greater trophic effects based on migration and angiogenesis.

## Abbreviations

BDNF: Brain-derived neurotrophic factor
bFGF: Basic fibroblast growth factor
BrdU: Bromodeoxyuridine
CM: Conditioned medium
CXCL14: Chemokine (C-X-C motif) ligand 14
DIG: Digoxigenin
DMEM: Dulbecco’s modified Eagle’s medium
FBS: Fetal bovine serum
G-CSF: Granulocyte colony-stimulating factor
GOT: Glutamic-oxaloacetic transaminase
HRP: Horseradish peroxidase
HUVEC: Human umbilical vein endothelial cell
IL: Interleukin
LAS AF: Leica Application Suite Advanced Fluorescence
MCP1: Monocyte chemotactic protein 1
MSC: Mesenchymal stem cell
NPY: Neuropeptide Y
PBS: Phosphate-buffered saline
PCNA: Proliferating cell nuclear antigen
RECA1: Rat endothelial cell antigen 1
RT-PCR: Reverse transcription-polymerase chain reaction
SCID: Severe combined immunodeficiency
SP: Side population
TRH-DE: Thyrotropin-releasing hormone-degrading enzyme
VE: Vascular endothelial
VEGF: Vascular endothelial growth factor
