# Erratum to: CXCL14 and MCP1 are potent trophic factors associated with cell migration and angiogenesis leading to higher regenerative potential of dental pulp side population cells

**DOI:** 10.1186/s13287-016-0346-8

**Published:** 2016-06-27

**Authors:** Yuki Hayashi, Masashi Murakami, Rei Kawamura, Ryo Ishizaka, Osamu Fukuta, Misako Nakashima

**Affiliations:** Department of Dental Regenerative Medicine, Center of Advanced Medicine for Dental and Oral Diseases, National Center for Geriatrics and Gerontology, Research Institute, Morioka 7-430, Obu, Aichi 474-8511 Japan; Department of Pediatric Dentistry, School of Dentistry, Aichi-Gakuin University suemoridouri 2-11, Nagoya, Aichi 464-8651 Japan; Department of Gerodontology, School of Dentistry, Aichi-Gakuin University suemoridouri 2-11, Nagoya, Aichi 464-8651 Japan

## Erratum

Following the publication of our article in *Stem Cell Research & Therapy* [1], we have become aware that errors were introduced inadvertently in Fig. 2.

**Fig. 2:**
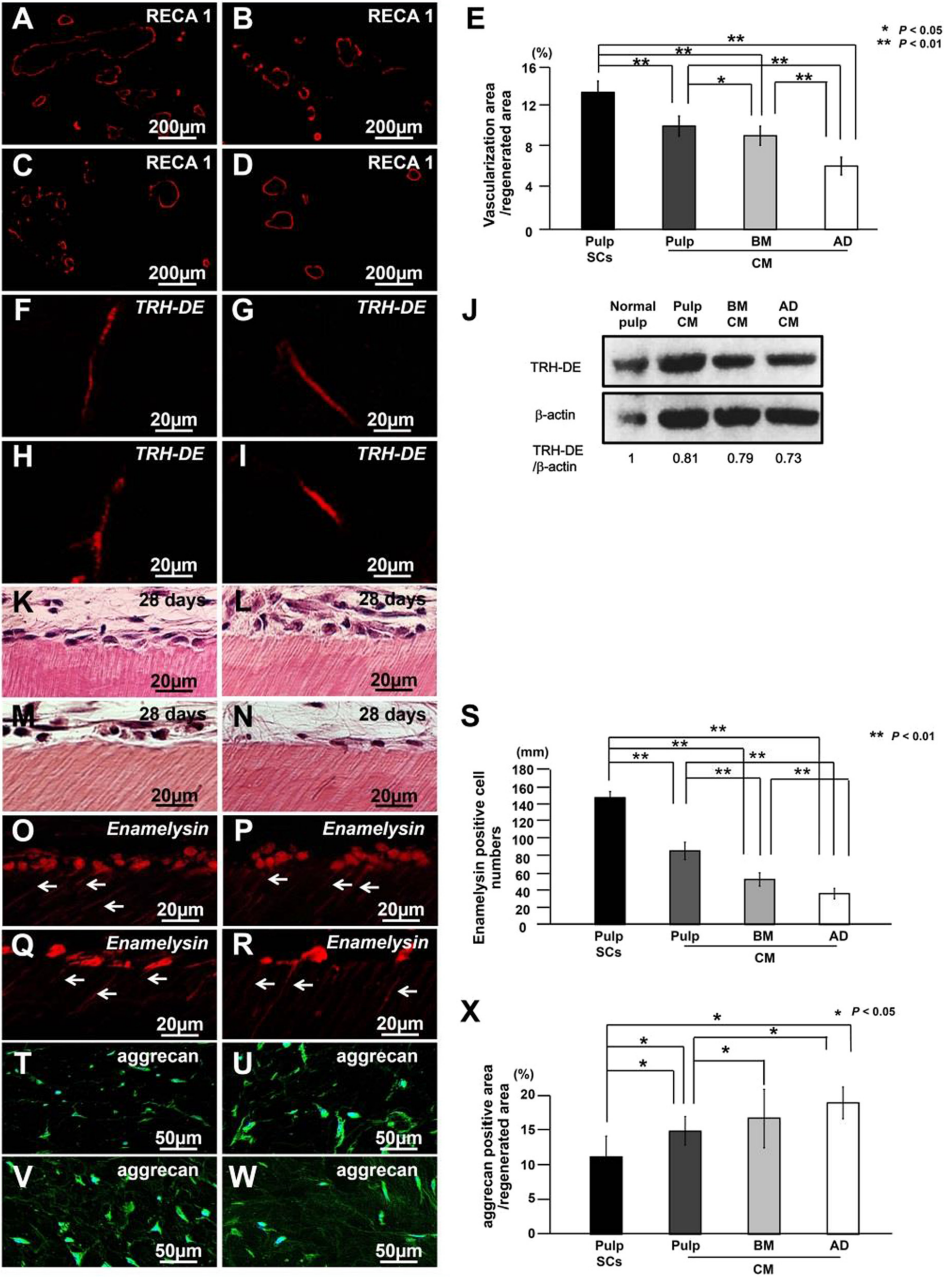
Characterization of regenerated tissue on day 28 in an ectopic tooth root transplantation model. **a**, **f**, **k**, **o**, **t** Transplant of pulp CD31^−^ side population (SP) cells (Pulp SCs). **b**, **g**, **l**, **p**, **u** Transplant of conditioned medium (CM) from pulp CD31^−^ SP cells (Pulp CM). **c**, **h**, **m**, **q**, **v** Transplant of CM from bone marrow CD31^−^ SP cells (BM CM). **d**, **i**,**n**, **r**, **w** Transplant of CM from adipose CD31^−^ SP cells (AD CM). **a**-**d** Immunostaining with rat endothelial cell antigen 1 (RECA1). **e** Ratio of vascularization area to the total regenerated area. (**f**-**i**) In situ hybridization analysis of expression of thyrotropin-releasing hormone-degrading enzyme (*TRH-DE*) as a pulp marker using an anti-sense probe reactive to both porcine and mouse genes. **j** Protein expression of TRH-DE in regenerated pulp after transplantation of CM from pulp, bone marrow (BM), and adipose (AD) CD31^−^ SP cells. **k**-**s** Odontoblastic differentiation potential in the regenerated pulp. **k**-**n** Odontoblastic cells along with the dentinal wall. **o**-**r** In situ hybridization analysis of *enamelysin*. Odontoblastic process extending into the tubular dentin (arrows). **s** Comparison of the numbers of *enamelysin*-positive cells along the dentinal wall. **t**-**w** Immunostaining with aggrecan (green) merged with Hoechst 33342 (Blue). **x** Ratio of aggrecan-positive area to the total regenerated area. Data are expressed as mean ± standard deviation of four determinations. *P < 0.05, **P < 0.01

During the preparation of panel J, the bands of β-actin were cut out and attached in the middle to remove non-specific bands. The bands of all samples were located on the same membrane. Unfortunately this panel was included in our article by mistake.

We are now providing a new version of Fig. 2 below which presents in panel J experimental data which were obtained at the same time.

The protein band intensity was re-quantified by densitometry (CS Analyzer) using the correct TRH-DE and β-actin band. Relative protein expression level was evaluated on the basis of band intensity of TRH-DE/β-actin. Each expression level of normal pulp was defined as 1.0.

We apologize for this error and confirm that the conclusions of the article are not affected.
